# Effects of Affordable Care Act on uninsured hospitalization: Evidence from Texas

**DOI:** 10.1111/1475-6773.14334

**Published:** 2024-06-03

**Authors:** Nima Khodakarami, Benjamin Ukert

**Affiliations:** ^1^ Department of Health Policy and Administration Penn State University Monaca Pennsylvania USA; ^2^ Department of Health Policy and Management Texas A&M University, School of Public Health College Station Texas USA

**Keywords:** ACA, hospital payor mix, uninsured hospitalization

## Abstract

**Objective:**

To examine the impact of the Affordable Care Act (ACA) health insurance exchanges (Marketplace) on the rate of uninsured discharges in Texas.

**Data Source and Study Setting:**

Secondary discharge data from 2011 to 2019 from Texas.

**Study Design:**

We conducted a retrospective study estimating the effects of the ACA Marketplace using difference‐in‐difference regressions, with the main outcome being the uninsured discharge rate. We stratified our sample by patient's race, age, gender, urbanicity, major diagnostic categories (MDC), and emergent type of admissions.

**Data Collection/Extraction Methods:**

We used Texas hospital discharge records for non‐elderly adults collected by the state of Texas and included acute care hospitals who reported data from 2011 to 2019.

**Principal Findings:**

The expansion of insurance through ACA Marketplaces led to reductions in the uninsured discharge rate by 9.9% (95% CI, −17.5%, −2.3%) relative to the baseline mean. The effects of the ACA were felt strongest in counties with any share of Hispanic, in counties with a larger population of Black, and other racial groups, in counties with a significant share of female and older age individuals, in counties considered to be urban, in high‐volume diagnoses, and emergent type of admissions.

**Conclusions:**

These findings indicate that the ACA facilitated a shift in hospital payor mix from uninsured to insured.

**What is known on this topic**

- The Affordable Care Act (ACA) increased insurance coverage; however, the impact of the ACA has not been well studied in non‐Medicaid expansion states.
- Disparities in health insurance take‐up have been documented. However, less is known about disparities in hospital care in nonexpansion states, and specifically Texas.

**What this study adds**

- The study provides evidence that the ACA Marketplace has increased insured hospital stays in Texas.
- The effects of the ACA were felt strongest in counties with large shares of individuals who identify as Hispanic, Black, women, and older age individuals.
- The study shows that uninsured hospital discharge rate dropped especially for high‐volume diagnosis that contributed to over 200 counts (~40%) of quarterly hospital discharges.

## 1 INTRODUCTION

Hospitals have a long history of being the insurer of last resort for individuals without health insurance. About 40 million uninsured nonelderly individuals received uncompensated care in hospitals valued at a cost of $44.9 billion in 2013 alone.^1^ Unfortunately, hospital profit margins are small and high levels of uncompensated care can have substantial negative consequences for hospitals, especially for rural hospitals, including the decision to close the hospital.^2^, ^3^ The main 2014 provisions of the Affordable Care Act (ACA), the establishment of individual Marketplace exchanges and state Medicaid expansions, increased insurance coverage, especially for low‐income individuals, and decreased the number of uninsured hospital stays, and could have thereby reduced a hospitals' share of uncompensated care and financial strain.^4^, ^5^, ^6^, ^7^, ^8^, ^9^, ^10^

The purpose of this paper is to examine the effect of the national components of the ACA health insurance expansion on the rate of uninsured and privately insured discharges in Texas. We used the universe of discharge data from 2011 to 2019 and implemented a difference‐in‐difference (DD) design, with the differences coming from the time of the implementation of the ACA and the pre‐ACA hospital uninsured discharge rate. This “bite” strategy has been used in the literature to identify policy effects where the policy was implemented nationally or regionally, and where a natural comparison group is not available.^11^, ^12^, ^13^, ^14^

From a policy perspective, Texas is an important state to study for several reasons. First, Texas is the second largest state in the nation but has the highest percentage of uninsured residents among all 50 states. Second, most studies have focused on identifying the effect of the Medicaid expansion on uninsured discharges, but the equally important and large expansion of the individual Marketplace has gained much less attention, even though more than 7% of all nationwide individuals Marketplace enrollees in 2014 gained coverage in Texas.^15^, ^16^, ^17^ Third, Texas is geographically, racially, and ethnically diverse. Texas has a large number of rural areas and rural hospitals that treat a high percentage of uninsured individuals. Thus, the effect of the ACA should have differentially impacted hospitals than in other states and could have led to especially strong coverage gains for non‐White residents.^18^ However, very little is known about the composition of insurance payor status for those with a hospital stay in Texas after the ACA.

Our study contributes on three margins relative to the existing literature. First, the understanding of the contribution of the Marketplace is underdeveloped, most likely because such analyses require the analyses of state‐specific discharge datasets, which can be expensive to purchase, and because of the large number of states that expanded Medicaid, which limits the identification of only the Marketplace effects. To date, earlier research on the ACA's impact on uncompensated care provision at hospitals focused mainly on evaluating the effects of Medicaid expansion and provided little focus on the effects felt in nonexpansion states.^19^, ^20^, ^21^, ^22^, ^23^ For example, in Massachusetts, a similar reform as the ACA reduced uninsured hospital discharges by 36% relative to its initial level and caused a reduction in the financial burden of uncompensated care.^24^, ^25^ Rigorous analyses on the effect of the Marketplace alone are needed to understand the healthcare behavior of individuals who are not considered to be poor enough to qualify for Medicaid. Additionally, our analysis can be useful to compare Marketplace results with Medicaid expansion studies to establish whether previous Medicaid expansion effects are symmetric when compared with those attributed to the Marketplace. However, such comparison does have caveats, as the Medicaid expansion affects individuals who may have different medical conditions and healthcare needs than individuals who qualify for coverage through Marketplace coverage.

Second, it is important to study nonexpansion states because it is of particular interest to understand the patient demographics and specific conditions that are most commonly treated for the working adult population after gaining coverage. The best knowledge we have on patient mix to date comes from Medicaid studies that only identifies the characteristics and attributes of new Medicaid patients.^26^ We contribute by providing detailed results on the working age population and provide subgroup analyses, revealing how the ACA Marketplace in Texas may have reduced racial disparities in uninsured hospital stays among working adults.

Lastly, most studies relied on hospital data from 2011 to 2015. We contribute to this literature by providing comprehensive evidence on the individual Marketplace contribution by changing the payor mix for 6 years following the ACA enactment (2014–2019).

## 2 DATA

We obtained the universe of inpatient hospital discharges from 2011 to 2019 Texas Health Care Information Collection (THCIC) Public Use Data File (PUDF).^27^ The data contain discharge information on all patients in state licensed hospitals and include a provider file identifying the facility. This timeframe gives us 3 years of pre‐ACA data and 6 years of post‐ACA data.

We created two hospital‐level outcome variables, one measuring the quarterly rate of uninsured discharges and the second measuring the quarterly rate of privately insured discharges, both as a share of all hospitalizations in each hospital. We identified insurance status based on prior studies and defined the uninsured as those with payer sources of self‐pay or charity care.^28^ Private coverage included the following sources of payment: managed care insurance (e.g., PPO, HMO, POS, and EPO), indemnity plans, commercial, and Blue Cross.

We limited our sample to patients between 18 and 64 and made several restrictions to the final analytical sample to focus on a panel of acute care hospitals with complete data for the entire study period. Specifically, we excluded nonacute care hospitals from the data, as the files include long‐term care, pediatric, and other hospitals. Thus, retaining only hospitals that self‐report to be acute care hospitals. A number of Texas hospitals opened and closed across our sample period, and we limited the sample to hospitals that were open for the full sample period. Nevertheless, our sample is a good representation of acute care hospitals who remained open for the 9 years in Texas and includes close to 100% of open acute care hospitals based on a comparison with American Hospital Association survey data. After these sample restrictions, the final sample consisted of 224 acute care hospitals over our time period. The hospitals were located across 77 counties that collectively encompass 78% of the Texas population.

A critical variable for our identification strategy was the average hospital uninsured discharge rate in the pre‐ACA period. Following earlier work by Courtemanche et al.^11^ and others that identified the effect of the national components of the ACA, including the exchanges, we aimed to capture the differential “dose” effect of the ACA on each hospital, where hospitals with higher uninsurance rates prior to the ACA were expected to see a larger treatment effect of the ACA compared with hospitals with lower uninsurance rates.^11^, ^12^, ^13^, ^14^ To do so, we calculate the 2013 mean uninsurance rate for each hospital as the independent variable of interest, which was 13.5%. The 25% and 75% quantiles for the variable in year 2013 were 7.8% and 17.6%, respectively. We also measured the effect of the ACA on the uninsured discharge rate using a binary indicator separating hospitals based on whether they were above or below median 2013 uninsurance rate (12.4%).

We included a large number of hospital‐quarter level control variables that we collected from the hospital discharge records. The demographic variables included the share of patient's falling into one of the following age groups (18–24, 25–34, 35–44, 45–54 and 55–64, with share of 18–24 being the reference group), gender, and race/ethnicity (which was required to be collected from providers per Texas law and included non‐Hispanic White, non‐Hispanic Black, and other, with share of non‐Hispanic White being the reference group). We also included two clinical variables including types of admission and severity of illness. Types of admission include elective, urgent, and emergency. An elective admission refers to hospital stays in which the patient's condition does not require immediate attention. An urgent admission refers to a situation where the patient's physical or mental disorder requires immediate attention for care and treatment. An emergency admission refers to a situation where the patient's medical condition is severe, life‐threatening, or potentially disabling and is in need of immediate medical intervention and care.^29^ We used the share of elective stays as the reference group. We further included the share of patients admitted based on severity of illness at the time of admission that reflects how severely the patient's body functions have deteriorated due to the illness. The patient's severity of illness is defined by four severity levels—minor, moderate, major, and extreme (with the shares of minor being the reference group). A patient is assigned to one of four severity levels depending on the number and interaction of complications and comorbidities for their specific base 3M All Patient Refined Diagnostic Related Groups inpatient grouper (3M APR DRG).^30^

We also included additional county‐level demographic and economic variables that were obtained from the U.S. Census Bureau's American Community Survey (ACS).^31^ The demographic controls included county‐level shares on age groups (18–24, 25–34, 35–44, 45–54, and 55–64, with the share of 18–24 being the reference group), gender, and race/ethnicity (non‐Hispanic White, non‐Hispanic Black, Hispanic, and non‐Hispanic other, with share of non‐Hispanic White being the reference group), citizenship (citizen and foreign born, with the share of foreign born being the reference group), and family controls including marital status (never married, now married, separated, widowed, and divorced, with the share of never married being the reference group), and the number of children in the household (with the share of households without children under the age of 18 being the reference group). ACS economic controls included education level (high school degree, some college, and college graduate, graduate degree, with the share of less than high school being the reference group), employment rate, and average household income grouped in four categories (below $50K, $50K–$100K, $100K–$150K, and $150K and more, with >$150K being the reference group). We also included the share of Medicaid enrollees as a control to account for potential woodwork effects.

Table 1 presents the summary statistics of the control variables obtained from the hospital stay on age, sex, self‐reported race and ethnicity, type of admission, and illness severity. Out of all discharges, 49% of the discharged patients were older than 44 years, 67% were female, 63% were identified as White, 41% were admitted for an elective reason, 41% were admitted with minor severity of illness, and 37% with a moderate severity of illness. Patient characteristics remained relatively stable before and after ACA.

**TABLE 1:** Means and standard deviations of study variables for hospital discharges: Pre‐and post‐Affordable Care Act (ACA).

	Pre‐ACA		Post‐ACA	
	Mean	SD	Mean	SD
Demographic controls				
Age and gender				
Age 18–24	0.148	0.123	0.134	0.121
Age 25–34	0.209	0.099	0.211	0.109
Age 35–44	0.154	0.040	0.149	0.040
Age 45–54	0.206	0.070	0.195	0.067
Age 55–64	0.283	0.117	0.311	0.134
Female	0.671	0.110	0.661	0.111
Race/ethnicity				
Non‐Hispanic White	0.631	0.254	0.665	0.259
Non‐Hispanic Black	0.119	0.110	0.126	0.114
Hispanic	0.257	0.251	0.277	0.263
Other	0.249	0.243	0.209	0.247
Clinical controls				
Type of admission				
Elective	0.406	0.260	0.396	0.261
Urgent	0.162	0.212	0.139	0.197
Emergency	0.432	0.244	0.466	0.243
Illness severity				
Minor	0.409	0.124	0.378	0.127
Moderate	0.369	0.065	0.385	0.065
Major	0.205	0.118	0.214	0.114
Extreme	0.086	0.118	0.086	0.116

*Note*: Summary statistics for inpatient stay characteristics using data from the 2011 to 2019 Texas hospital discharge data are displayed for the 2011–2013 (pre‐ACA) and 2014–2019 (post‐ACA) periods.

Abbreviation: SD, standard deviation.

We present the summary statistics of county‐level control variables obtained from the ACS in Table S1. The average county population had about 17% of adults aged 55–64, 66% were older than 24 years, 50% were female, 46% were non‐Hispanic White, and 38% Hispanic. About 85% were US citizens, 50% were married, 13% had a child under 18, 55% had some college or a higher level of education, 92% were employed, and nearly 50% had income below $50,000. Table S1 exhibits population characteristics within sample counties that remained similar before and after the ACA.

**FIGURE 1:**
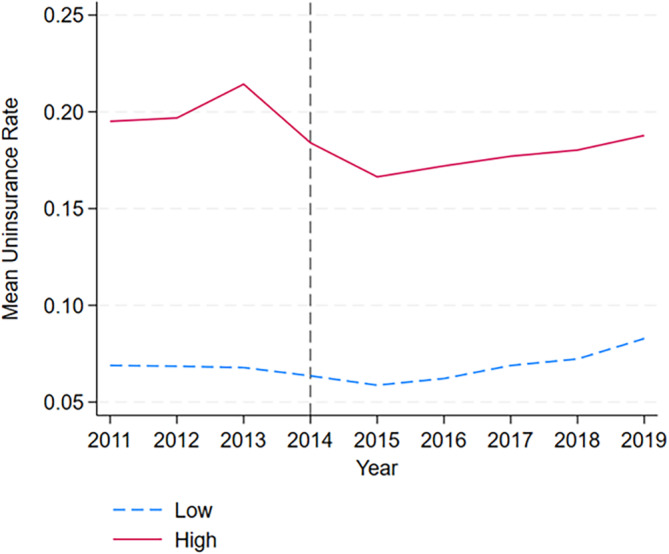
Time trends of mean uninsurance rate for hospitals with high and low rates of uninsured discharges. Figure displays the trend in uninsured discharge rate for hospitals with the above‐median and below‐median uninsurance rates based on the 2013 uninsurance rate of 13.5%.

Figure 1 displays the average uninsured rate across the sample period for hospitals stratified into high‐ and low‐average pretreatment uninsured rate based on the 2013 uninsurance rate. The pretreatment trends appear relatively similar across both groups prior to the ACA, but after the ACA, we see a larger decrease in the uninsurance rate for hospitals with a high baseline uninsured discharge rate. The trend turns positive in 2016, but the level remains below the pre‐ACA period. The observed trend is consistent with the findings of the Center on Budget and Policy Priorities, which showed an increase in the uninsurance rate in 2016 and later.^32^

## 3 METHOD

We used a DD approach to estimate the impact of the ACA in Texas on the insurance status. Specifically, we formalized the ACA dose response effect in the following regression:

(1) $$y_{\mathrm{ht}}=\gamma_{0}+\gamma_{1}\left(2013\text{Uninsure}d_{h}*\mathrm{Pos}t_{t}\right)+\gamma_{2}X_{\mathrm{ht}}+\tau_{\tau }+\alpha_{h}+\varepsilon_{\mathrm{ht}},$$

where $y_{\mathrm{ht}}$ represents the uninsured/private discharge rate for hospital h in quarter t in the period 2011–2018. $2013\text{Uninsure}d_{h}$ represents the 2013 average uninsured discharge rate for each hospital h, which implies that a hospital with a zero uninsured discharge rate would experience no effect of the ACA, yet the effect would increase linearly as the hospital uninsured rate rises. $\mathrm{Pos}t_{t}$ is an indicator equal to one (indicating the ACA implementation) starting in 2014 and zero otherwise. $X_{\mathrm{ht}}$ is a vector of discharge and ACS control variables, $\tau_{\tau }$ is a year fixed effect, $\alpha_{h}$ is a hospital fixed effect, and $\varepsilon_{\mathrm{ht}}$ is the error term. Heteroscedasticity robust standard errors clustered at the hospital level were used.

We also measured the effect of the ACA on the uninsured discharge rate using a binary indicator separating hospitals based on whether they were above or below median 2013 uninsurance rate. This approach generalized the result from Equation (1) to a binary indicator. To do so, we estimate the following regression model:

(2) $$y_{\mathrm{ht}}=\gamma_{0}+\gamma_{1}\left(2013\text{MedianUninsure}d_{h}*\mathrm{Pos}t_{t}\right)+\gamma_{2}X_{\mathrm{ht}}+\tau_{\tau }+\alpha_{h}+\varepsilon_{\mathrm{ht}},$$

where $2013\text{MedianUninsure}d_{h}$ is equal to 1 if the 2013 hospital's uninsured rate is above the median rate and zero otherwise. The remaining variables were the same as described above.

We then estimated an event study model to indirectly evaluate the assumption of common pretreatment trends that infer whether treated hospitals would have followed a similar trend had they not been treated. To estimate this, we used the interaction of 2013 uninsurance rate with year dummies to trace out the ACA's impact for each year. The regression takes the following form, with 2013 being the omitted reference year:

(3) $$y_{\mathrm{ht}}=\begin{array}{l} \gamma_{0}+\gamma_{1}\left(2013{\text{Uninsured}}_{h}*\text{Year}2011\right)\\ +\;\gamma_{2}\left(2013{\text{Uninsured}}_{h}*\text{Year}2012\right)\\ +\;\gamma_{3}\left(2013{\text{Uninsured}}_{h}*\text{Year}2014\right)\\ +\;\gamma_{4}\left(2013{\text{Uninsured}}_{h}*\text{Year}2015\right)\\ +\;\gamma_{5}\left(2013{\text{Uninsured}}_{h}*\text{Year}2016\right)\\ +\;\gamma_{6}\left(2013{\text{Uninsured}}_{h}*\text{Year}2017\right)\\ +\;\gamma_{7}\left(2013{\text{Uninsured}}_{h}*\text{Year}2018\right)+\gamma_{8}X_{\mathrm{ht}}+\tau_{\tau }+\alpha_{h}+\varepsilon_{\mathrm{ht}}. \end{array}$$

$2013\text{Uninsure}d_{h}$ represents the 2013 average uninsured discharge rate for each hospital h, and the year variables (Year2011etc.) are year dummies interacted with the hospital uninsurance rate in 2013. $X_{\mathrm{ht}}$ is a vector of controls described above, $\tau_{\tau }$ is a year fixed effect, $\alpha_{h}$ is a hospital fixed effect, and $\varepsilon_{\mathrm{ht}}$ is the error term.

We further tested whether our main findings are robust to slight changes in the hospital sample and whether the ACA may have led to changes in inpatient volume. We re‐estimated our main regression presented in the Equation (1) where we included all hospitals that self‐identified as an acute care facility in a minimum of one quarter of each year (*n* = 243). We also included hospitals that were self‐identified as an acute care facility at least in one quarter within the span of the study period (*n* = 264).

To identify subgroup effects, we cut the data based on ACS‐specific county information into two samples based on whether the hospital fell into a county with above versus below median of the characteristics. For example, for age, we separated the counties based on median county age. We repeated this process for different sample stratification and reported the results for both uninsured and privately insured outcomes. We also cut the hospitals based on whether they were located in a rural or urban county in accordance with the US Office of Management and Budget classification from the 2010 census.^33^ We identified 32 rural hospitals that accounted for 23% of the studied discharges. We also evaluated whether we saw changes in the type of hospital admissions based on the patients' Major Diagnostic Category (MDC). We display subsample results for MDCs with 200 or more discharges per quarter, which account for about 42% of hospital discharges. Lastly, we cut the sample by emergent and nonemergent admissions. We performed additional sensitivity analysis by performing the analysis at rating area. Moreover, we estimated Equation (1) where our outcome variable were the total discharge volume and the hospital‐level Medicaid inpatient rate. Finally, to account for woodwork effect, we used the county‐level Medicaid coverage obtained from the 1‐year ACS files once as a control variable and once as an outcome variable in Equation (1).

## 4 RESULTS

Table 2 shows the difference‐in‐differences estimates on the impact of the ACA on the uninsurance rate and the privately insured rate. In each panel, the first column reports full regression results with all control variables including the hospital and quarterly time fixed effects, patient characteristics, and the ACS county‐level variables. The results with piecemeal addition of control variables are presented in Table S2. In Table S3, we also display results where we perform the analysis at the rating area.

**TABLE 2:** The effect of the Affordable Care Act (ACA) marketplace on the inpatient uninsurance and private insurance rates.

	Uninsured inpatient rate	Privately insured inpatient rate
	Estimated change (SE)	Estimated change (SE)
Mean 2013 uninsurance rate	−0.099* (0.038)	0.089* (0.039)
Above‐median uninsurance rate	−0.017* (0.009)	0.015 (0.008)
Incl. PUDF and ACS controls	Yes	Yes

*Note*: Difference‐in‐differences regression results are displayed where we evaluate the effect of the ACA on the rates of hospitalization for the uninsured and privately insured. Results are also displayed for a binary independent indicator variable equal to 1 for the above‐median uninsurance rate in 2013 and zero otherwise. Each cell corresponds to the outcome from a separate regression model. Standard errors (SE) displayed in parentheses are heteroskedasticity‐robust and clustered at the hospital level. *** indicates statistical significance at 0.1% level, ** at 1% level, and * at 5% level.

Abbreviations: ACS, American Community Survey; PUDF, Texas Inpatient Public Use Data File.

We translate the coefficients into average effects based on the 2013 average hospital uninsured rate (13.5 percentage point). We find that the ACA reduced the hospital uninsurance rate by (0.099*0.135) 1.3 percentage points, or 9.9% relative to the baseline mean. In Appendix Figure S1 we display the effect along the distribution of the hospital uninsured rate. The results remain qualitatively similar when using the median uninsurance rate (1.7 percentage points). The estimate from specification that controls for county Medicaid coverage shares provide similar estimates. Turning to the privately insured, we observe that the ACA led to an increase in the insured hospitalized population by 1.2 percentage points or 2.4% relative to the baseline mean. The results are qualitatively similar using the binary treatment variable.

**FIGURE 2:**
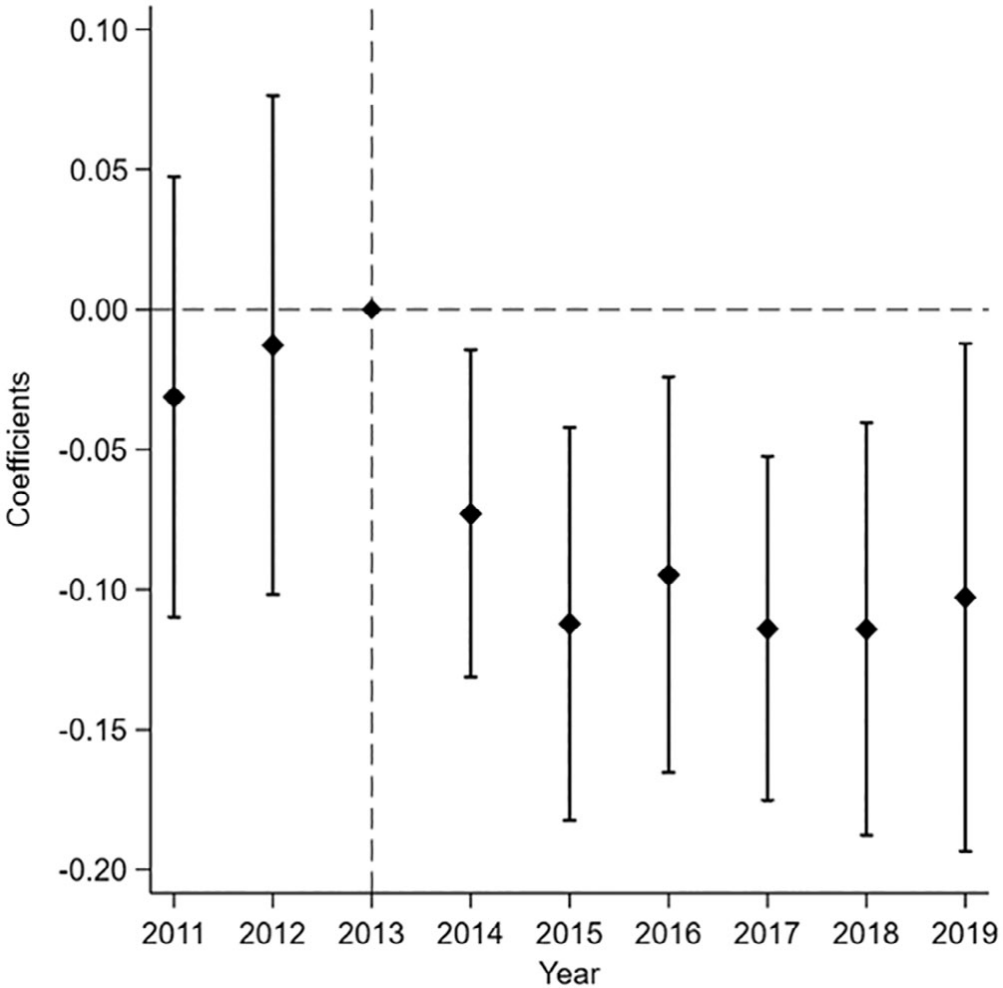
Event study coefficients trend in the pre and post‐Affordable Care Act, (reference year is 2013). Figure displays coefficients and 95% confidence intervals from the event study difference‐in‐differences regression where the outcome is the uninsured discharge rate. Standard errors are clustered at the hospital level.

Figure 2 displays the event study results for Equation (1). Similar to the descriptive Figure 1, we show similar pre‐trends prior to the ACA in 2014 and diverging trends in the post‐period. The coefficient estimates in the post‐treatment years display an immediate effect of the ACA in 2014 that grows in 2015 and remains consistent throughout 2019. Table S4 of the Appendix includes the coefficients.

### 4.1 Subsample analysis

Table 3 reports results for different sample stratifications based on the ACS data and the rural/urban classification. The findings in the last column with all patient and county controls imply a significant impact of the ACA on uninsured hospital discharges in counties with populations of individuals who identified as Hispanic, and in counties with above‐median populations of non‐Hispanic Black and those identifying as neither non‐Hispanic Black nor non‐Hispanic White. For example, our findings indicate a significant 0.92 (0.067*0.138) percentage points decrease, or 6.7% relative decrease in counties with above‐median Hispanic population, and 1.2 percentage points drops in counties with below‐median Hispanic population. For individuals identifying as Black and neither non‐Hispanic Black nor White, the effects were 0.9 and 1.1 percentage points respectively. In counties with above‐median age (39.5 years), the ACA reduced the rate of uninsured hospitalization by (0.067*0.136) 0.91 percentage points or 6.7%. The effect of the ACA was also concentrated in counties with above‐median population share of women, indicating a 1.1 percentage point (0.081*0.137) or 8.1% reduction. Lastly, urban counties saw reductions, experiencing a 1.1 percentage points (0.085*0.132) or 8.5% drops. Subsample analyses using the private insurance rate as an outcome suggest similar trends for the specific subsamples. These findings are outlined in Table S5 of the Appendix and display similarly significant effects for the same subsamples.

**TABLE 3:** Subsample effects of the Affordable Care Act (ACA) marketplace on the uninsurance rates.

		Uninsured inpatient rates estimated change (SE)		
	Only PUDF demographic controls	Add PUDF clinical controls	Add ACS demographic controls	Add ACS economic controls
Race/ethnicity				
Above‐median Hispanic, sample size: 5436, mean 2013 Hispanic uninsured rate = 0.138				
Mean 2013 uninsurance rate	−0.054* (0.027)	−0.068* (0.025)	−0.069* (0.025)	−0.067* (0.025)
Below‐median Hispanic, sample size: 2628, mean 2013 Hispanic uninsured rate = 0.124				
Mean 2013 uninsurance rate	−0.082* (0.038)	−0.081* (0.036)	−0.094* (0.035)	−0.103** (0.036)
Above‐median Black, sample size: 5544, mean 2013 Black uninsured rate = 0.128				
Mean 2013 uninsurance rate	−0.065** (0.022)	−0.067** (0.019)	−0.067*** (0.017)	−0.067*** (0.017)
Below‐median Black, sample size: 2520, mean 2013 Black uninsured rate = 0.151				
Mean 2013 uninsurance rate	−0.058 (0.054)	−0.079 (0.043)	−0.067 (0.049)	−0.067 (0.049)
Above‐median White, sample size: 2772, mean 2013 White uninsured rate = 0.152				
Mean 2013 uninsurance rate	−0.045 (0.050)	−0.058 (0.044)	−0.050 (0.051)	−0.052 (0.048)
Below‐median White, sample size: 5292, mean 2013 White uninsured rate = 0.126				
Mean 2013 uninsurance rate	−0.074** (0.023)	−0.078*** (0.019)	−0.079*** (0.016)	−0.079*** (0.017)
Above‐median other race, sample size: 6552, mean 2013 other race uninsured rate = 0.135				
Mean 2013 uninsurance rate	−0.075* (0.029)	−0.085** (0.024)	−0.086** (0.025)	−0.086** (0.025)
Below‐median other race, Sample size: 1512, Mean 2013 Other Race Uninsured rate = 0.138				
Mean 2013 uninsurance rate	−0.010 (0.034)	−0.011 (0.033)	−0.033 (0.030)	−0.033 (0.027)
Age				
Above‐median age, sample size: 4680, counties median population age in 2013 = 39.5, mean 2013 uninsured rate = 0.136				
Mean 2013 uninsurance rate	−0.061* (0.028)	−0.064* (0.029)	−0.063* (0.029)	−0.067* (0.030)
Below‐median age, sample size: 3384, mean 2013 uninsured rate = 0.134				
Mean 2013 uninsurance rate	−0.043 (0.031)	−0.045 (0.030)	−0.049 (0.030)	−0.051 (0.029)
Gender				
Above‐median female, sample size: 6912, counties median population female rate = 0.50, mean 2013 uninsured rate = 0.137				
Mean 2013 uninsurance rate	−0.071** (0.026)	−0.080** (0.022)	−0.082*** (0.022)	−0.081*** (0.022)
Below‐median female, sample size: 1152, mean 2013 uninsured rate = 0.121				
Mean 2013 uninsurance rate	−0.011 (0.074)	−0.014 (0.069)	−0.019 (0.064)	−0.017 (0.062)
Rural/urban				
Urban, sample size: 6876, mean 2013 uninsured inpatient rate = 0.132				
Mean 2013 uninsurance rate	−0.080*** (0.024)	−0.082*** (0.022)	−0.085*** (0.021)	−0.085*** (0.021)
Rural, sample size: 1188, mean 2013 uninsured inpatient rate = 0.161				
Mean 2013 uninsurance rate	−0.014 (0.080)	−0.012 (0.074)	−0.046 (0.096)	−0.047 (0.109)

*Note*: Subsample regression results are displayed from Equation (1), where we evaluate the effect of the ACA on the uninsured discharge rates. Each subsample was stratified based on the median county‐level characteristic. Each cell corresponds to the outcome from a separate difference‐in‐differences regression model. Standard errors (SE) displayed in parentheses are heteroskedasticity‐robust and clustered at the hospital level. *** indicates statistical significance at 0.1% level, ** at 1% level, and * at 5% level.

Abbreviations: ACS, American Community Survey; PUDF, Texas Inpatient Public Use Data File.

Table S6 displays the results for the most common MDCs based on their total number of hospitalizations. On average, MDCs 14 (pregnancy, childbirth, and puerperium), 5 (circulatory system), 4 (respiratory system), and 6 (digestive system) had average quarterly discharges of 391, 305, 210, and 205 per hospital, respectively. Relative to the average effect in Table 2, we observe similar large declines in the uninsurance rate across all MDCs. We observe reduction in uninsured deliveries (1.1 percentage points or 19%), circulatory system (1.4 percentage points or 14%), respiratory system (1.0 percentage points or 12%), and digestive system (1.0 percentage points or 8.5%).

Table S7 presents results for the sample stratified by emergent and nonemergent admission. The findings suggest a 1.5 percentage points decrease in emergent admissions or 7.3% drop relative to baseline mean.

### 4.2 Robustness checks and volume analyses

The ACA may have impacted the number of hospitalizations, if previously uninsured individuals were more willing to engage with the healthcare system. Table S8 evaluated whether the ACA led to a change in inpatient volume. Across all regression specifications, we did not find changes in inpatient volume. Table S9 presents the effect of the ACA on the county share of individuals with Medicaid coverage and the Medicaid discharge rate. Table S10 presents the outcomes for the extended samples and suggests that the ACA reduced the hospital uninsurance rate by 1.1 percentage points, or 9.5% relative to the baseline means, or by 0.9 percentage points, or 7.9%, for the largest sample. In summary, the results were quite similar to our main regression, indicating our primary findings were robust to change in the hospital sample.

## 5 DISCUSSION

The main purpose of this study was to determine whether expanding insurance coverage through the ACA Marketplace reduced the rate of uninsured care provided by hospitals in Texas. We observed decreases in the rate of uninsured discharges and increases in the insured discharge rate. These findings indicate that the ACA facilitated a shift in hospital payor mix from the uninsured to insured. Effects were generally concentrated in counties with vulnerable groups. Analysis on hospital volume displays that the ACA did not increase the demand for hospital care, as the number of hospitalizations remained constant.

The effects of the ACA were felt strongest in counties with a larger population of Hispanic, Black, and other racial groups, and in counties with above‐median share of female and older age individuals. These findings reiterate previous work, which showed that the uninsurance rate dropped especially for individuals identified as Black and Hispanic above the decrease observed for those identified as White.^11^, ^14^, ^34^, ^35^, ^36^, ^37^, ^38^, ^39^, ^40^, ^41^ Our findings are also consistent with Texas studies that showed that Hispanics and those aged 50–64 have made significant gains in insurance coverage after 2013.^42^, ^43^ A recent associational report also displayed that the rate of uninsured hospital discharges decreased strongly for urban counties, and our subsample findings align with that report.^44^

Study results also provide results stratified by inpatient diagnoses. Decreases in uninsured discharges were equally distributed in the top four conditions that accounted for 40% of all discharges (related to pregnancy, circulatory, respiratory, and digestive system). These findings are important as some of these conditions are coming with high insurance payments, especially in the case of circulatory care.

Study results also provide fresh evidence relative to what is known on the impact of the ACA on the uninsured discharge rate. Relative to work focusing on the effect of the ACA Medicaid expansion, which found reductions between 30% and 72%, our findings for the Marketplace alone are smaller in magnitude (~10%). Early evidence from only the 2006 Medicaid expansion in Massachusetts displayed a reduction of 36% of uninsured non‐Medicare inpatient stays. A national analysis by Nikpay et al.^19^ evaluated the change in uninsured discharges in the first two quarters of 2014 and found a reduction of uninsured discharges by 72% in Medicaid expansion states. Singer et al. found a 30% reduction after the ACA through 2016; however, this included Medicaid expansion and nonexpansion states.^45^ Results in Texas may be smaller compared with other work because Texas's uninsurance rate remained relatively high even after the implementation of the Marketplace, thus leading to only a marginal reduction in the uninsured discharge rate. Further, changes in hospital care is driven by individuals who are either working or live in households with moderate incomes, as such, one could expect that this population is healthier than those who qualify for Medicaid, which could explain the attenuated effect. In sum, it seems that the Texas Marketplace has a smaller effect than those reported in Medicaid expansion studies; however, only a limited number of studies has evaluated the impact on uninsured discharges, which limits our comparison.

The study has several limitations. This analysis relied on data from Texas, as such, our findings may not generalize to other states. Texas is special in the sense that a large share of the population was uninsured prior to the ACA and that the Texas ACA Marketplace is one of the largest. Further, differences in demographic mix across states and medical needs can influence the take‐up of insurance and the need for hospital care. As such, similar analysis should be performed for other non‐Medicaid expansion states, such as Florida. Further, our analysis cannot speak to the recent impact of the changes in the ACA Marketplace premium policy changes that occurred after 2018. At the same time, one may expect that the patient mix in hospitals changed substantially during COVID‐19, as such, our findings should not be thought of representative during that time period.

Future research should continue to study how the ACA Marketplace has impacted hospitals’ long‐term payor mix and financial well‐being. Moreover, the pandemic led to substantial increases in Medicaid enrollment, even in non‐Medicaid expansion states, and more work should be done to understand how this has affected hospital payor mix during the COVID‐19 period.^46^

## FUNDING INFORMATION

No funding to report.

## CONFLICT OF INTEREST STATEMENT

The authors declare no conflicts of interest.

## Supporting information


**Data S1.** Supporting information.
