# Supplementary material for: Effects of Affordable Care Act on uninsured hospitalization: Evidence from Texas
Source: Health Serv Res. 2024 Jun 3;59(4):e14334. doi: 10.1111/1475-6773.14334 (PMC11249825; doi:10.1111/1475-6773.14334)
Supplement: Supplementary file 1 — Data S1. Supporting information. [file HESR-59-0-s001.docx]

**Appendix**

**Figure S1:** The Effect of the ACA Marketplace on Uninsurance Rates of Hospitalization at Pre-Treatment Uninsured Rate.


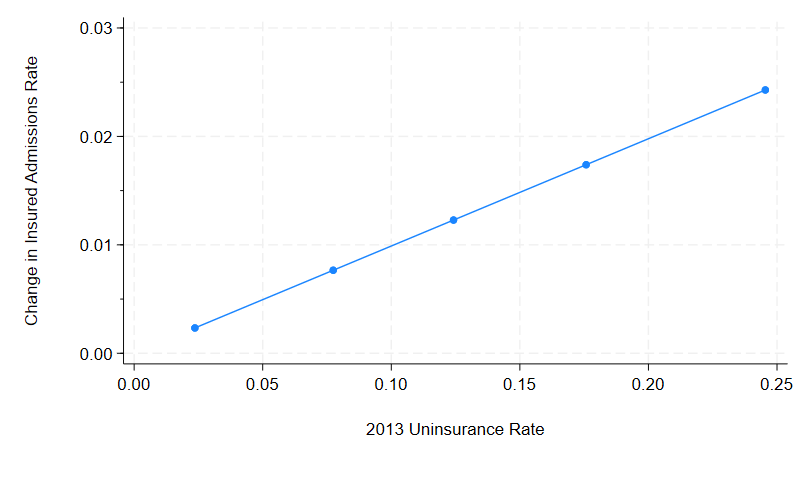


*Note:* Figure displays the effect along the distribution on the effect of the ACA (2013 uninsurance rate) within the 10 and 90 percentiles. ACA=Affordable Care Act.

**Table S1**: Summary Statistics of the ACS Population Characteristics in Sample Counties: Pre- and Post-ACA

|  | Pre-ACA | | Post-ACA | |
| --- | --- | --- | --- | --- |
|  | *Mean* | *SD* | *Mean* | *SD* |
| **Demographic controls**  *Age and Gender* | | | | |
| Age 18-24 | 0.168 | 0.043 | 0.167 | 0.042 |
| Age 25-34 | 0.228 | 0.025 | 0.232 | 0.025 |
| Age 35-44 | 0.219 | 0.024 | 0.216 | 0.023 |
| Age 45-54 | 0.217 | 0.020 | 0.207 | 0.020 |
| Age 55-64 | 0.168 | 0.028 | 0.178 | 0.027 |
| Female | 0.504 | 0.014 | 0.503 | 0.014 |
| *Race/ethnicity* | | | | |
| Non-Hispanic white | 0.460 | 0.189 | 0.441 | 0.187 |
| Non-Hispanic Black | 0.107 | 0.074 | 0.108 | 0.074 |
| Hispanic | 0.377 | 0.213 | 0.389 | 0.212 |
| Non-Hispanic-Other | 0.055 | 0.036 | 0.061 | 0.041 |
| *Place of born* |  |  |  |  |
| U.S. citizen | 0.848 | 0.077 | 0.844 | 0.076 |
| *Family controls* |  |  |  |  |
| Married | 0.503 | 0.053 | 0.491 | 0.049 |
| Household with child under 18 | 0.134 | 0.012 | 0.129 | 0.012 |
| **Economic controls** |  | | |  |
| *Education* |  |  |  |  |
| Less than high school degree | 0.207 | 0.070 | 0.193 | 0.067 |
| High school degree | 0.253 | 0.051 | 0.252 | 0.052 |
| Some College | 0.293 | 0.035 | 0.294 | 0.035 |
| College graduate | 0.249 | 0.088 | 0.262 | 0.091 |
| *Employment* |  |  |  |  |
| Employed | 0.923 | 0.014 | 0.935 | 0.016 |
| Unemployed | 0.077 | 0.014 | 0.065 | 0.017 |
| *Income* |  | | |  |
| Income below 50 | 0.493 | 0.098 | 0.462 | 0.098 |
| Income 50-100 | 0.296 | 0.029 | 0.296 | 0.024 |
| Income 100-150 | 0.123 | 0.037 | 0.134 | 0.035 |
| Income 150+ | 0.089 | 0.045 | 0.108 | 0.053 |

*Note:* Summary statistics for county population characteristics using data from the 2011-2019 American Community Survey (ACS) 5-year estimate are displayed for the 2011-2013 (pre-ACA) and 2014-2019 (post-ACA) periods. SD=Standard deviation. ACA=Affordable Care Act.

**Table S2:** The Effect of the ACA Marketplace on the Inpatient Uninsurance and Private Insurance Rates

|  | **Panel I** | | | |  | **Panel II** | | | |  |
| --- | --- | --- | --- | --- | --- | --- | --- | --- | --- | --- |
|  | Uninsured inpatient rates | | | |  | Privately insured inpatient rates | | | |  |
|  | Only PUDF Demographic Controls | Add PUDF Clinical Controls | | Add ACS Demographic Controls | Add County Medicaid coverage control | Only PUDF Demographic Controls | Add PUDF Clinical Controls | | Add ACS Demographic Controls | Add County Medicaid coverage control |
|  |  |  | |  |  |  |  | |  |  |
| Mean 2013 Uninsurance Rate | -0.073*  (0.034) | -0.075*  (0.034) | | -0.087*  (0.038) | -0.098*  (0.042) | 0.068*  (0.034) | 0.076*  (0.037) | | 0.082*  (0.040) | 0.092*  (0.036) |
|  |  | |  | |  |  | |  | |  |
|  |  |  | |  |  |  |  | |  |  |
| Above Median Uninsurance Rate | -0.017*  (0.009) | -0.017*  (0.008) | | -0.017*  (0.008) | -0.016*  (0.008) | 0.013  (0.007) | 0.015  (0.008) | | 0.015  (0.008) | 0.017*  (0.008) |
|  |  | |  | |  |  | |  | |  |
|  |  |  | |  |  |  |  | |  |  |
| Hospital and Time fixed effects | YES | YES | | YES | YES | YES | YES | | YES | YES |
| Incl. PUDF Demographic Controls | YES | YES | | YES | YES | YES | YES | | YES | YES |
| Incl. PUDF Clinical Controls | NO | YES | | YES | YES | NO | YES | | YES | YES |
| Incl. ACS Demographic Controls | NO | NO | | YES | YES | NO | NO | | YES | YES |

*Note*: Difference in differences regression results are displayed where we evaluate the effect of the ACA Marketplace on the rate of hospitalization for the uninsured and privately insured. Results are also displayed for a binary independent indicator variable equal to 1 for the above median uninsurance rate in 2013 and zero otherwise. Each cell corresponds to the outcome from a separate regression model. Standard errors (SE) displayed in parentheses are heteroskedasticity-robust and clustered at the hospital level. *** indicates statistical significance at 0.1% level, ** at 1% level, and * at 5% level. PUDF= Texas Inpatient Public Use Data File. ACS= American Community Survey. ACA=Affordable Care Act.

**Table S3:** The Effect of the ACA Marketplace on the Rating Area Uninsurance Inpatient Rates

|  | Only PUDF Demographic Controls | Add PUDF Clinical Controls | Add ACS Demographic Controls | Add ACS Economic Controls |
| --- | --- | --- | --- | --- |
| *All Rating Area 2022: Sample size:936, Rating area’s 2013 mean uninsured rate= 0.100* | | | | |
| Mean 2013 Uninsurance Rate | -0.256*  (0.109) | -0.257*  (0.109) | -0.259*  (0.109) | -0.268*  (0.108) |

*Note*: Results are effects of the ACA on the rates of hospitalization at the rating area. Each cell corresponds to the outcome from a separate regression model. Standard errors (SE), heteroskedasticity-robust and clustered at the rating area, are displayed in parentheses. *** indicates statistical significance at 0.1% level, ** at 1% level, and * at 5% level. PUDF= Texas Inpatient Public Use Data File. ACS= American Community Survey. ACA=Affordable Care Act.

**Table S4:** Event Study Coefficient Results Table

|  | Coefficient, with all controls | Robust St. Errors |
| --- | --- | --- |
| Uninsured Discharge Rate for Year 2013 ×Year 2011 | -0.031 | 0.045 |
| Uninsured Discharge Rate for Year 2013 ×Year 2012 | -0.013 | 0.048 |
| Uninsured Discharge Rate for Year 2013 ×Year 2014 | -0.073* | 0.029 |
| Uninsured Discharge Rate for Year 2013 ×Year 2015 | -0.112** | 0.035 |
| Uninsured Discharge Rate for Year 2013 ×Year 2016 | -0.095** | 0.035 |
| Uninsured Discharge Rate for Year 2013 ×Year 2017 | -0.114** | 0.031 |
| Uninsured Discharge Rate for Year 2013 ×Year 2018 | -0.114** | 0.037 |
| Uninsured Discharge Rate for Year 2013 ×Year 2019 | -0.103* | 0.045 |

*Note:* Results are coefficients from the event study regression of equation (2). Robust St. Errors=Standard errors, heteroskedasticity-robust and clustered at the hospital. *** indicates statistical significance at 0.1% level, ** at 1% level, and * at 5% level.

**Table S5**: Subsample Effect of the ACA Marketplace on the Private Insurance Rates

|  | Only PUDF Demographic Controls | Add PUDF Clinical Controls | Add ACS Demographic Controls | Add ACS Economic Controls |
| --- | --- | --- | --- | --- |
| **Race/Ethnicity** | | | | |
| *Above median Hispanic, Sample size: 5,436, Mean 2013 Hispanic Uninsured rate=0.138* | | | | |
| Mean 2013 Uninsurance Rate | 0.050  (0.040) | 0.050  (0.040) | 0.084*  (0.040) | 0.083*  (0.041) |
| *Below median Hispanic, Sample size: 2,628, Mean 2013 Hispanic Uninsured rate=0. 124* | | | | |
| Mean 2013 Uninsurance Rate | 0.018  (0.051) | 0.022  (0.049) | 0.040  (0.048) | 0.047  (0.052) |
| *Above median Black, Sample size: 5,544, Mean 2013 Black Uninsured rate=0.128,* | | | | |
| Mean 2013 Uninsurance Rate | 0.037  (0.032) | 0.039  (0.033) | 0.041  (0.029) | 0.044  (0.030) |
| *Below median Black, Sample size: 2,520, Mean 2013 Black Uninsured rate=0.151* | | | | |
| Mean 2013 Uninsurance Rate | 0.107  (0.072) | 0.107  (0.072) | 0.104  (0.072) | 0.096  (0.071) |
| *Above median White, Sample size: 2,772, Mean 2013 White Uninsured rate=0.152* | | | | |
| Mean 2013 Uninsurance Rate | 0.040  (0.033) | 0.049  (0.035) | 0.050  (0.031) | 0.053  (0.032) |
| *Below median White, Sample size: 5,292, Mean 2013 White Uninsured rate=0.126* | | | | |
| Mean 2013 Uninsurance Rate | 0.090  (0.047) | 0.092*  (0.045) | 0.093*  (0.043) | 0.090*  (0.042) |
| *Above median other race, Sample size: 6,552, Mean 2013 Other Race Uninsured rate=0.135* | | | | |
| Mean 2013 Uninsurance Rate | 0.063  (0.039) | 0.094*  (0.040) | 0.095*  (0.039) | 0.095*  (0.040) |
| *Below median other race, Sample size: 1,512, Mean 2013 Other Race Uninsured rate=0.138* | | | | |
| Mean 2013 Uninsurance Rate | 0.054  (0.040) | 0.053  (0.037) | 0.032  (0.030) | 0.039  (0.034) |
| **Age** |  |  |  |  |
| *Above median Age, Sample size: 4,680, Mean 2013 Uninsured rate=0.136* | | | | |
| Mean 2013 Uninsurance Rate | 0.058  (0.033) | 0.062*  (0.031) | 0.069*  (0.033) | 0.073*  (0.034) |
| *Below median Age, Sample size: 3,384, Mean 2013 Uninsured rate=0.134* | | | | |
| Mean 2013 Uninsurance Rate | 0.054  (0.050) | 0.057  (0.050) | 0.066  (0.054) | 0.066  (0.054) |
| **Gender** |  |  |  |  |
| *Above median Female, Sample size: 6,912, Mean 2013 Uninsured rate=0.137* | | | | |
| Mean 2013 Uninsurance Rate | 0.047  (0.031) | 0.065*  (0.029) | 0.069*  (0.029) | 0.071*  (0.030) |
| *Below median Female, Sample size: 1,152, Mean 2013 Uninsured rate=0.121* | | | | |
| Mean 2013 Uninsurance Rate | 0.058  (0.086) | 0.068  (0.071) | 0.058  (0.078) | 0.069  (0.075) |
| **Rural/Urban** |  |  |  |  |
| *Urban, Sample size: 6,876, Mean 2013 uninsured inpatient rate= 0.132* | | | | |
| Mean 2013 Uninsurance Rate | 0.052  (0.032) | 0.057*  (0.029) | 0.061*  (0.029) | 0.062*  (0.030) |
| *Rural, Sample size: 1,188, Mean 2013 uninsured inpatient rate= 0.161* | | | | |
| Mean 2013 Uninsurance Rate | 0.082  (0.105) | 0.075  (0.101) | 0.157  (0.094) | 0.150  (0.098) |

*Note:* Difference in differences regression results are displayed where we evaluate the effect of the ACA on the rates of hospitalization for the privately insured subsample population. Each cell corresponds to the outcome from a separate regression model. Standard errors (SE), heteroskedasticity-robust and clustered by hospital, are in parentheses. *** indicates statistical significance at 0.1% level, ** at 1% level, and * at 5% level. PUDF= Texas Inpatient Public Use Data File. ACS= American Community Survey. ACA=Affordable Care Act.

**Table S6**: The Effect of ACA the Marketplace on Uninsurance Rates for MDCs with Average Quarterly Discharge of 200 or More

|  | Only PUDF Demographic Controls | Add PUDF Clinical Controls | Add ACS Demographic Controls | Add ACS Economic Controls |
| --- | --- | --- | --- | --- |
| *MDC 14: Pregnancy, Childbirth and Puerperium, Sample size: 6,486, 2013 Uninsured rate= 0.060* | | | | |
| Mean 2013 Uninsurance Rate | - 0.187***  (0.051) | -0.186***  (0.052) | -0.191***  (0.053) | -0.189***  (0.052) |
| *MDC 5: Circulatory System, Sample size: 7,565, 2013 Uninsured rate= 0.095* | | | | |
| Mean 2013 Uninsurance Rate | -0.101**  (0.037) | -0.096*  (0.037) | -0.122**  (0.046) | -0.144**  (0.046) |
| *MDC 4*: *Respiratory System, Sample size: 7,414, 2013 Uninsured rate= 0.085* | | | | |
| Mean 2013 Uninsurance Rate | -0.076  (0.045) | -0.080*  (0.045) | -0.107*  (0.056) | -0.120*  (0.056) |
| *MDC 6*: *Digestive System, Sample size: 7,701, 2013 Uninsured rate= 0.122* | | | | |
| Mean 2013 Uninsurance Rate | -0.059*  (0.028) | -0.061*  (0.029) | -0.074*  (0.034) | -0.085*  (0.035) |

*Note:* Difference in differences regression results are displayed where we evaluate the effect of the ACA on the rates of hospitalization for the subsample population. Each cell corresponds to the outcome from a separate regression model. Standard errors (SE), heteroskedasticity-robust and clustered by hospital, are in parentheses. *** indicates statistical significance at 0.1% level, ** at 1% level, and * at 5% level. PUDF= Texas Inpatient Public Use Data File. ACS= American Community Survey. ACA=Affordable Care Act. MDC=Major Diagnostic Categories.

**Table S7**: The Effect of the ACA Marketplace on Uninsurance Rates Based on Emergent and Non-emergent Admissions

|  | Only PUDF Demographic Controls | Add PUDF Clinical Controls | Add ACS Demographic Controls | Add ACS Economic Controls |
| --- | --- | --- | --- | --- |
| *Emergent: Sample size: 8,064, Mean 2013 emergent uninsured rate= 0.204* | | | | |
| Mean 2013 Uninsurance Rate | -0.070***  (0.019) | -0.075***  (0.017) | -0.074***  (0.018) | -0.073***  (0.019) |
| *Nonemergent: Sample size:8,064, Mean 2013 non-emergent uninsured rate=0.075* | | | | |
| Mean 2013 Uninsurance Rate | -0.099  (0.059) | -0.070  (0.044) | -0.073  (0.45) | -0.072  (0.044) |

*Note:* Difference in differences regression results are displayed where we evaluate the effect of the ACA on the rates of hospitalization for the subsample population. Each cell corresponds to the outcome from a separate regression model. Standard errors (SE), heteroskedasticity-robust and clustered by hospital, are in parentheses. *** indicates statistical significance at 0.1% level, ** at 1% level, and * at 5% level. PUDF= Texas Inpatient Public Use Data File. ACS= American Community Survey. ACA=Affordable Care Act.

**Table S8:** The Effect of the ACA Marketplace on Inpatient Discharges

|  | Only PUDF Demographic Controls | Add PUDF Clinical Controls | Add ACS Demographic Controls | Add ACS Economic Controls | Add County Medicaid coverage Control |
| --- | --- | --- | --- | --- | --- |
| *Dependent variable: Count of a hospital’s quarterly discharges; Mean discharge rate=2,631* | | | | | |
| Mean 2013 Uninsurance Rate | -193  (358) | -153  (382) | -164  (378) | -162  (383) | -226  (364) |

*Note:* Difference in differences regression results are displayed where we evaluate the effect of the ACA on the volume of hospital discharges. Each cell corresponds to the outcome from a separate regression model. Standard errors (SE), heteroskedasticity-robust and clustered by hospital, are in parentheses. *** indicates statistical significance at 0.1% level, ** at 1% level, and * at 5% level. PUDF= Texas Inpatient Public Use Data File. ACS= American Community Survey. ACA=Affordable Care Act.

**Table S9**: The Effect of the ACA Marketplace on Medicaid Coverage and Medicaid Inpatient Rates

|  | Only PUDF Demographic Controls | Add PUDF Clinical Controls | Add ACS Demographic Controls | Add ACS Economic Controls |
| --- | --- | --- | --- | --- |
| ***Panel I:*** *Dependent variable: ACS Medicaid coverage; Mean ACS Medicaid Coverage=0.108, sample size=7,164* | | | | |
| Mean 2013 Uninsurance Rate | -0.096  (0.073) | -0.099  (0.072) | -0.067  (0.072) | -0.065  (0.070) |
| ***Panel II:*** *Dependent Variable: Medicaid inpatient rate; Mean Medicaid insured inpatient rate=0.195, sample size=8,064* | | | | |
| Mean 2013 Uninsurance Rate | 0.035  (0.023) | 0.029  (0.023) | 0.026  (0.023) | 0.026  (0.023) |

*Note:* Difference in differences regression results are displayed where we evaluate the effect of the ACA on the rate of Medicaid coverage at county level and Medicaid inpatient rate. Each cell corresponds to the outcome from a separate regression model. Standard errors (SE), heteroskedasticity-robust and clustered by hospital, are in parentheses. *** indicates statistical significance at 0.1% level, ** at 1% level, and * at 5% level. PUDF= Texas Inpatient Public Use Data File. ACS= American Community Survey. ACA=Affordable Care Act.

**Table S10**: The Effect of the ACA Marketplace on Uninsurance Inpatient Rates

|  | Only PUDF Demographic Controls | Add PUDF Clinical Controls | Add ACS Demographic Controls | Add ACS Economic Controls | Add Medicaid coverage Control |
| --- | --- | --- | --- | --- | --- |
| ***Panel I****: Dependent variable: Uninsured inpatient rate; Mean 2013 uninsured inpatient rate =0.126, Median 2013 uninsured inpatient rate =0.118; Sample size= 8,748* | | | | | |
| Mean 2013 Uninsurance Rate | -0.064*  (0.031) | -0.064*  (0.030) | -0.081*  (0.038) | -0.095*  (0.037) | -0.102*  (0.041) |
| Above Median Uninsurance Rate | -0.009*  (0.004) | -0.009*  (0.005) | -0.010*  (0.005) | -0.011*  (0.005) | -0.010*  (0.005) |
| ***Panel II****: Dependent variable: Uninsured inpatient rate; Mean 2013 uninsured inpatient rate =0.119, Median 2013 uninsured inpatient rate =0.111; Sample size= 9,504* | | | | | |
| Mean 2013 Uninsurance Rate | -0.059*  (0.030) | -0.058*  (0.030) | -0.070*  (0.035) | -0.079*  (0.038) | -0.085*  (0.042) |
| Above Median Uninsurance Rate | -0.013*  (0.007) | -0.013*  (0.007) | -0.015*  (0.008) | -0.015*  (0.008) | -0.015  (0.009) |

*Note*: Difference in differences regression results are displayed where we evaluate the effect of the ACA on the rates of hospitalization for the uninsured on extended samples. Results are also displayed for a binary independent indicator variable equal to 1 for the above median uninsurance rate in 2013 and zero otherwise. Each cell corresponds to the outcome from a separate regression model. Standard errors (SE) displayed in parentheses are heteroskedasticity-robust and clustered at the hospital level. *** indicates statistical significance at 0.1% level, ** at 1% level, and * at 5% level. PUDF= Texas Inpatient Public Use Data File. ACS= American Community Survey. ACA=Affordable Care Act.
